# Long non-coding RNA MAGI2*-AS3* inactivates STAT3 pathway to inhibit prostate cancer cell proliferation via acting as a microRNA-424-5p sponge

**DOI:** 10.7150/jca.60749

**Published:** 2022-01-01

**Authors:** Xin Wei, Yi Hou, Yan Zhang, Huaiwei Zhang, Zhou Sun, Xiangdi Meng, Zhixin Wang

**Affiliations:** 1 Department of Urology, China-Japan Union Hospital of Jilin University, 126 Xiantai Street Changchun 130033, P.R.China; 2 Department of Endocrinology, China-Japan Union Hospital of Jilin University, 126 Xiantai Street Changchun 130033, P.R.China

**Keywords:** *MAGI2-AS3*, prostate cancer, miR-424-5p, STAT3

## Abstract

Aberrant expression of long non-coding RNAs (lncRNAs) that results in sustained activation of cell growth promoting pathways is an important mechanism in driving prostate cancer progression. In the present study, we explored differentially expressed lncRNAs in two microarray datasets of prostate benign and malignant tissues. We found that *MAGI2-AS3* was one of the most downregulated lncRNAs in prostate tumors, which was further confirmed in our collected clinical samples. The function assays showed that *MAGI2-AS3* overexpression decreased cell viability and led to obvious cell apoptosis in PC-3 and DU145 prostate cancer cells. Elevation of *MAGI2-AS3* decreased the activity of STAT3 in PC-3 and DU145. In addition, microRNA-424-5p (miR-424-5p), a positive regulator of STAT3 pathway, was predicted as a target of *MAGI2-AS3*, furthermore, the interaction between *MAGI2-AS3* and miR-424-5p was confirmed via reverse-transcript polymerase chain reaction (RT-qPCR), dual luciferase reporter assay and RNA immunoprecipitation (RIP). *MAGI2-AS3* upregulated miR-424-5p and downregulated COP1 in PC-3 and DU145. More importantly, IL6-induced activation of STAT3 pathway could attenuate the biological effect of *MAGI2-AS3* in PC-3 and DU145. In clinical samples, *MAGI2-AS3* levels were negatively correlated with miR-424-5p expression, while positively correlated with *COP1* mRNA expression. Altogether, the current study revealed *MAGI2-AS3* as a novel negative regulator of prostate cancer development.

## Introduction

Prostate cancer is the most prevalent cancer type for males worldwide, accounting for nearly 19% of newly diagnosed cancer cases in United States, 2018 1. In China, statistics suggest that the morbidity and mortality of prostate cancer has been gradually increased in recent years 2. As the primary treatment approach for patients with prostate cancer, androgen deprivation therapy has greatly improved the overall survival of the patients. However, acquired and *de novo* resistance to the inhibition of androgen receptor signaling occur in these patients, which is closely associated with the death of patients with prostate cancer 3. Therefore, it is important to study the molecular mechanisms of prostate cancer progression and provides novel targets and biomarkers for the treatment of patients.

Long non-coding RNAs (lncRNAs) are defined as single-stranded transcripts (more than 200 nucleotides) with no protein coding potential. Previously known as “junk” RNAs, recent studies have revealed that they play central roles in mediating normal physiology processes and the development of human diseases. Mechanistically, lncRNAs can act as microRNA (miRNA) sponge, directly interact with miRNAs, leading to downregulation of miRNAs and upregulation of miRNA targets. Most recent reports have identified several oncogenic or tumor suppressor lncRNAs in prostate cancer via the miRNA sponge method 4-6. For example, UCA1 (urothelial carcinoma associated 1) promotes prostate cancer progression via binding to miR-143 and increase MYO6 expression 4. MEG3 enhances expression of QKI-5 via interaction with miR-9-5p in prostate cancer cells, therefore triggering cell apoptosis and inhibiting cell proliferation and motility 5. The expression of *MAGI2-AS3*, which locates on chromosome 7q21.11, is associated with several cancer types such as breast cancer and bladder cancer 7-9. A recent bioinformatic analysis shows that, *MAGI2-AS3* in combination with 2 lncRNAs and 6 mRNAs, are signatures of prognostic prediction of patients with prostate cancer 10. The precise molecular mechanisms and functions of *MAGI2-AS3* have not been studied in prostate cancer yet.

In current study, we discovered that *MAGI2-AS3* is a significantly downregulated lncRNA in prostate cancer according to previously published datasets, which was further confirmed in our collected clinical samples. In prostate cancer cell lines, we showed that *MAGI2-AS3* inhibited cell proliferation and promoted cell apoptosis. In addition, *MAGI2-AS3* sponged miR-424-5p, a miRNA with oncogenic potential, and inactivated STAT signaling in the studied cells. The data collectively demonstrated a tumor suppressor role of *MAGI2-AS3* in prostate cancer.

## Materials and methods

### Collection of patient samples

109 pairs of prostate tumors and adjacent non-tumor tissues were collected during surgery in China-Japan Union Hospital of Jilin University from July 2016 to June 2019. Inclusion criteria: Patients diagnosed with prostate cancer and has provided informed consents. Exclusion criteria: Patients received chemotherapy or other therapy before the surgery. These patients were aged from 45 to 82 with the average age of 66. The protocol of the current study was reviewed and approved by the Ethical Committee of China-Japan Union Hospital of Jilin University. These tissues were stored in -80℃ before subjected to RNA extraction.

### Cell culture and treatment

The immortalized prostate myofibroblast stromal cell line WPMY-1, prostate cancer cell lines PC-3 and DU145 were obtained from ATCC (Manassas, VA). These cells were maintained in DMEM (Gibco; Thermo Fisher Scientific) supplemented with 10% FBS (Hyclone; Thermo Fisher Scientific). The culture condition was 5% CO_2_ and 37℃. Cells were treated with a mixture of IL6 (Sigma Aldrich) (10 ng/mL) or equal amount of vehicle (sterilized water) and the culture medium for 24 h. *MAGI2-AS3* (Gene ID: 100505881) was inserted into pcDNA3.1 for overexpression. miR-424-5p mimic and miR-NC mimic were purchased from GenePharma (Suzhou, China). Plasmids and miRNA mimic were transfected into cells by Lipofectamine 3000 reagent (Invitrogen; Thermo Fisher Scientific).

### RNA extraction and realtime PCR (RT-qPCR)

Nuclear and cytoplasm RNA was isolated by Cytoplasmic & Nuclear RNA Purification Kit (Norgen Biotek, Ontario, Canada) following manufacturer's protocol. Tissues and cells were both treated with TRIzol reagent (Invitrogen; Thermo Fisher Scientific, Carlsbad, CA) for the extraction of total RNA. RNA was reverse transcribed into cDNA via M-MLV Reverse Transcriptase (Invitrogen; Thermo Fisher Scientific). The RT-qPCR was carried out using Taq Pro Universal SYBR qPCR Master Mix (Vazyme, Nanjing, China). The protocol was as following: step 1: 95℃, 30 sec; step 2: 95℃, 5 sec; 60℃, 30 sec, 40 cycles. The primers were: Bcl2-F: 5'-GGTGGGGTCATGTGTGTGG-3'; Bcl2-R: 5'-CGGTTCAGGTACTCAGTCATCC-3'; Mcl1-F: 5'-TGCTTCGGAAACTGGACATCA-3'; Mcl1-R: 5'-TAGCCACAAAGGCACCAAAAG-3'; *MAGI2-AS3*-F: 5'-TGGGTCTGTGCAGAGTTGAG-3'; *MAGI2-AS3*-R: 5'-AGGGAGTCTAGGCCCCTTCT-3'; miR-424-5p-F: 5'-GCCGAGCAGCAGCAATTCAT-3'; miR-424-5p-R: 5'-CTCAACTGGTGTCGTGGA-3'; COP1-F: 5'-TGAGTGGCTTATACTCTCCTGTC-3'; COP1-R: 5'-AACGTGCTATTATACCAAGGCTG-3'; GAPDH-F: 5'-GGAGCGAGATCCCTCCAAAAT-3'; GAPDH-R: 5'-GGCTGTTGTCATACTTCTCATGG-3'; U6-F: 5'-CTCGCTTCGGCAGCACA-3'; U6-R: 5'-AACGCTTCACGAATTTGCGT-3'. We selected GAPDH and U6 as internal controls of mRNA and miRNA, respectively. The relative expression of each gene was calculated using the comparative ^ΔΔCt^ method.

### Protein extraction and western blotting

RIPA lysis buffer (Thermo Fisher Scientific) was selected for extraction of protein from cells. BCA kit was used to determine the protein concentration. 15 μg proteins were loaded on the SDS-PAGE gel, prior to being transferring to PVDF membrane. After blocking in 5% non-fat milk, membrane was incubated with the primary antibody and secondary antibody sequentially. The blots were developed with ECL Western Blotting Substrate (Pierce; Thermo Fisher Scientific). The antibodies were listed as follow: STAT3 (Cat.12640, 1:2000), p-STAT3 (Tyr707) (Cat.9145, 1:1000) and GAPDH (Cat.97166, 1:10000) antibodies were obtained from CST (Danvers, MA). COP1 (Cat.GTX34159, 1:2000) antibody was purchased from GeneTex (San Antonio, TX). HRP-conjugated goat anti-mouse (ab6789, 1:100000) and HRP-conjugated goat anti-rabbit (ab6721, 1:100000) were products of Abcam (Cambridge, UK).

### Bioinformatic analysis

The expression of *MAGI2-AS3* in benign prostate (n=14) and prostate cancer (n=36) was downloaded from GEO database (GSE46602). The expression of *MAGI2-AS3* in benign prostate (n=8) and prostate cancer (n=13) were downloaded from GEO database (GSE55945). The expression of *MAGI2-AS3* in 52 normal prostate tissues and 492 prostate tumors was retrieved from GTEx and TCGA-PRAD projects. The potential target miRNA of *MAGI2-AS3* was predicted by the miRDB software (http://mirdb.org/). Correlation between *MAGI2-AS3* with COP1 and STAT target genes was analyzed on the GEPIA software (http://gepia.cancer-pku.cn/).

### RNA immunoprecipitation (RIP)

The RIP assay was applied to study the interaction between *MAGI2-AS3* and miR-424-5p using the EZMagna RIP RNA-binding protein immunoprecipitation kit (Millipore) according to the manufacturer's instructions. Briefly, prostate cancer cells were lysed with RNA lysis buffer supplemented with protease and RNase inhibitors. The lysate was incubated with magnetic beads pre-coated with Ago2 antibody (Cat. 10686-1-AP, ProteinTech) or a control IgG (Cat. ab133470, Abcam) at 4°C overnight. Then, the RNAs were subjected to RT-qPCR to detect *MAGI2-AS3* and miR-424-5p expression.

### Determination of cell proliferation and cell apoptosis

The cell proliferation was assessed by the CCK-8 kit (AbMole, Houston, TX). Medium was mixed with CCK-8 solution, incubated with cells for 2 hours, then transferred to a new 96-well plate. OD 450 of each well was read by the Microplate Reader to manifest the cell number.

The percentage of apoptotic cells was detected by Annexin V-FITC/PI Apoptosis Detection Kit (Vazyme, Nanjing, China). Cells were collected and stained with Annexin V-FITC and PI sequentially. After that, cells were subjected to flow cytometry analysis and the data were analyzed with FlowJo software. Cells were distributed in 4 phases according to the PI and FITC status. FITC+/PI+ and FITC+/PI- cells were the apoptotic cells.

### Dual luciferase reporter assay

Full length of MAIG2-AS3 (*MAGI2-AS3*-WT) or the mutant *MAGI2-AS3* (*MAGI2-AS3*-Mut) was inserted into pmir-GLO plasmid. Cells were co-transfected with pmir-GLO*-MAGI2-AS3*-WT or pmir-GLO*-MAGI2-AS3*-Mut and miR-424-5p mimic or miR-NC mimic by Lipofectamine 3000 (Invitrogen; Thermo Fisher Scientific). After 48 hours, the relative luciferase of each group was detected by the Dual-Glo Luciferase Assay System (Promega, Madison, WI). pGMSTAT3-Luc (Yeason Biotech, Shanghai, China) containing 4 binding sites for STAT3 was transfected into cells. At 24 hours after treatment, the luciferase activity was detected by the Dual-Glo Luciferase Assay System to reflect STAT3 activity.

### Statistical analysis

All data were calculated and the graphs were generated by the GraphPad Prism 6.0. Student's t test was used to compare the differences between 2 groups. For the differences among 3 groups, they were analyzed by one-way ANOVA followed by Tukey test. Pearson analysis was selected to study the association between gene expression. All experiments were repeated three times. P value less than 0.05 was considered as statistically significant.

## Results

### *MAGI2-AS3* was a downregulated lncRNA in prostate cancer

**Figure 1:**
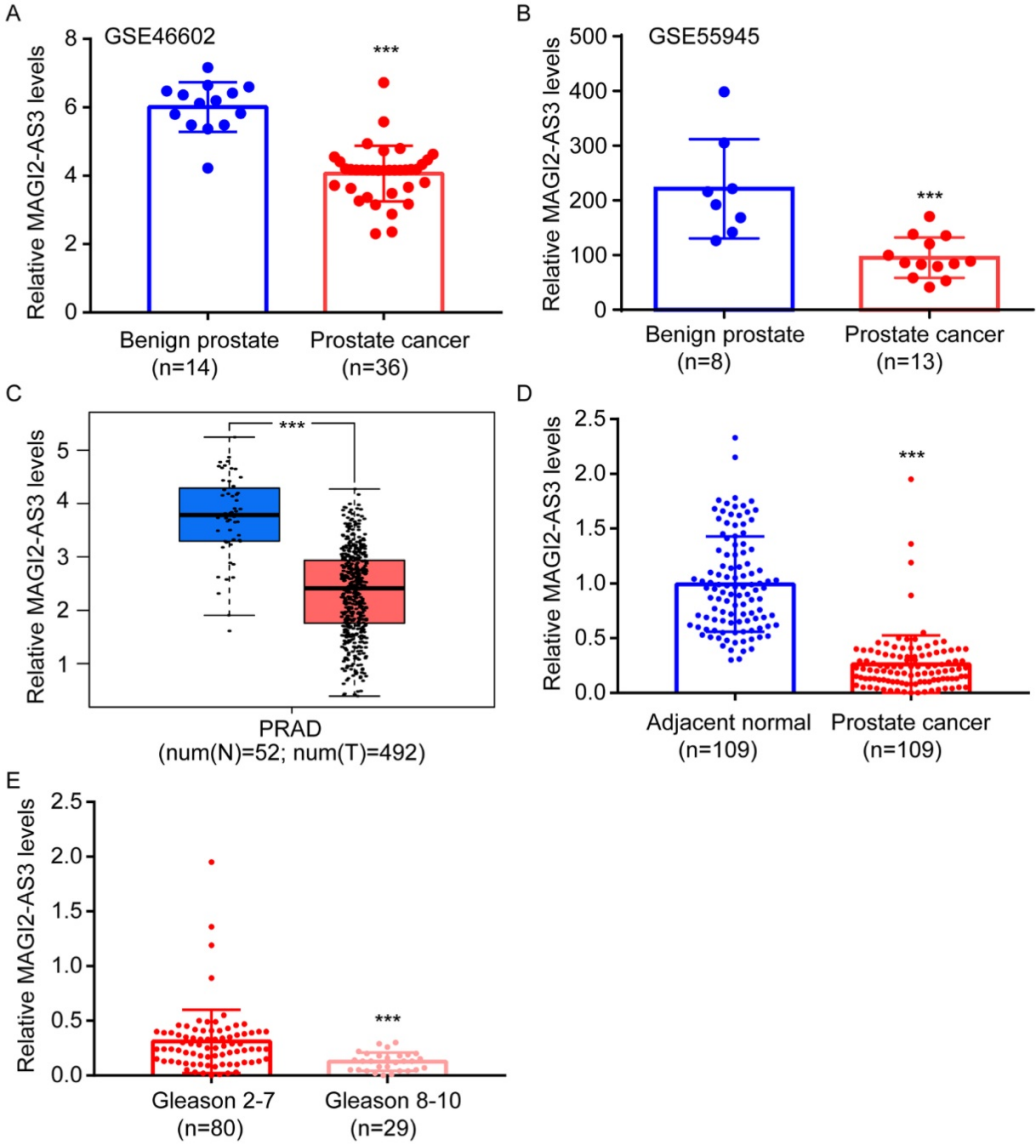
***MAGI2-AS3* was a downregulated lncRNA in prostate cancer.** A-B. Bioinformatic analysis of *MAGI2-AS3* expression in benign prostate and prostate cancer tissues from GSE46602 and GSE55945 datasets. C. Bioinformatic analysis of *MAGI2-AS3* expression in normal prostate and prostate cancer from TCGA dataset. D. RT-qPCR detection of *MAGI2-AS3* expression in 109 pairs of prostate cancer and adjacent normal prostate. E. Comparison of *MAGI2-AS3* expression between prostate tumors of high Gleason score and those of low Gleason score. ***, p<0.001.

To explore the differentially expressed lncRNAs in prostate cancer, we firstly retrieved expression profiles of prostate cancer and benign prostate from GSE46602 and GSE55945. *MAGI2-AS3* was found to be one of the most significantly downregulated lncRNA in prostate cancer from both GSE46602 and GSE55945 (Fig. 1A-B). For validation, we also analyzed *MAGI2-AS3* expression in TCGA-PRAD dataset. Consistently, compared with 52 normal prostate tissues, *MAGI2-AS3* was significantly decreased in 492 prostate cancers (Fig. 1C). We further collected patient samples. According to RT-qPCR results, *MAGI2-AS3* was approximately 3-fold lower in prostate cancer than in the adjacent normal tissues (Fig. 1D). Decreased *MAGI2-AS3* expression was associated with high Gleason score (Fig. 1E). *MAGI2-AS3* expression was not associated with age or tumor stage (Table 1).

### Overexpression of *MAGI2-AS3* inhibited prostate cancer cell proliferation

**Figure 2:**
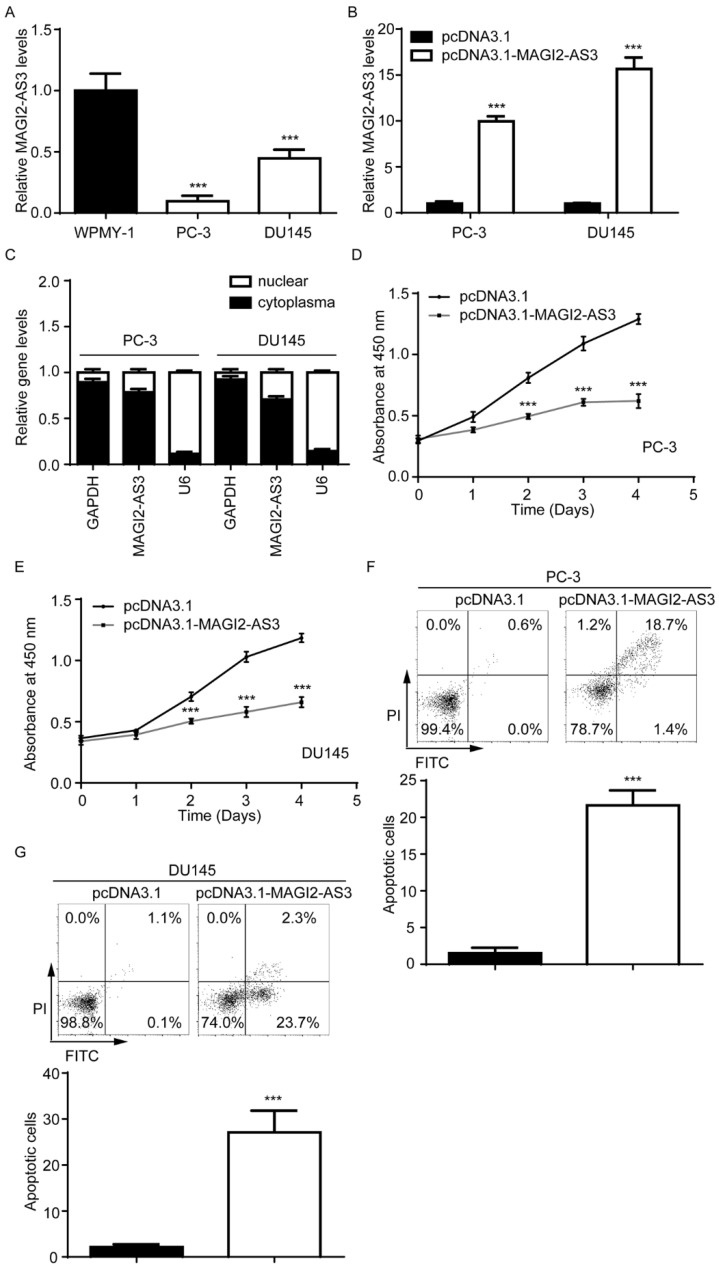
** Elevation of *MAGI2-AS3* inhibited cell proliferation and promoted cell apoptosis in prostate cancer cells.** A. *MAGI2-AS3* expression was detected in WPMY-1 and two prostate cancer cell lines (PC-3 and DU145) by RT-qPCR. B. *MAGI2-AS3* expression was detected in PC-3 and DU145 with transfection of pcDNA3.1 or pcDNA3.1-*MAGI2-AS3* by RT-qPCR. C. RT-qPCR was used to detect GAPDH mRNA, *MAGI2-AS3* and U6 expression in nuclear and cytoplasm fractions of PC-3 and DU145 cells. D-E. The cell proliferation ability was detected in PC-3 (D) and DU145 (E) cells with transfection of pcDNA3.1 or pcDNA3.1-*MAGI2-AS3*. F-G. The percentage of apoptotic cells was detected in PC-3 (F) and DU145 (G) cells with transfection of pcDNA3.1 or pcDNA3.1-*MAGI2-AS3*. ***, p<0.001.

We next detected *MAGI2-AS3* expression in two prostate cancer cell lines (PC-3 and DU145) and the immortalized prostate myofibroblast stromal cell line WPMY-1. *MAGI2-AS3* was decreased in PC-3 and DU145 compared with WPMY-1 (Fig. 2A). We cloned full length of *MAGI2-AS3* into pcDNA3.1 plasmid. After transfection, it was found that *MAGI2-AS3* expression was increased 9-fold and 15-fold in PC-3 and DU145 cells respectively (Fig. 2B). Via separating nuclear and cytoplasm fractions of cells, we found that *MAGI2-AS3* was mainly localized in cytoplasm of PC-3 and DU145 cells (Fig. 2C). Function assays showed that elevation of *MAGI2-AS3* inhibited cell proliferation of both PC-3 and DU145 cells (Fig. 2D-E). In addition, we detected the percentage of apoptotic PC-3 and DU145 cells by flow cytometry analysis. We found that *MAGI2-AS3* overexpression led to significant cell apoptosis in PC-3 and DU145 cells (Fig. 2F-G).

### *MAGI2-AS3* inactivated STAT signaling in prostate cancer cells

**Figure 3:**
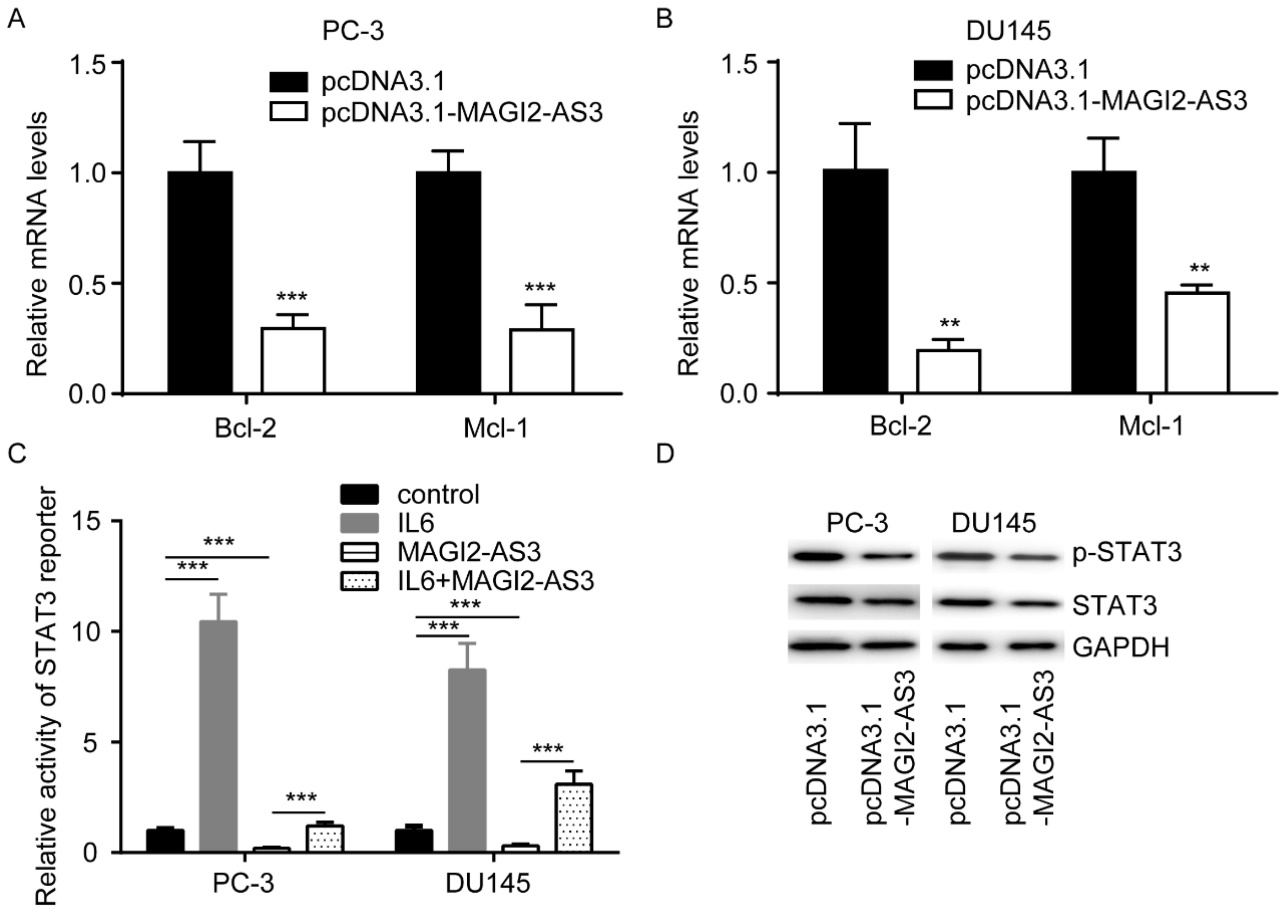
***MAGI2-AS3* inactivated STAT3 signaling in prostate cancer cells.** A-B. Bcl-2 and Mcl-1 mRNA levels were determined in PC-3 (A) and DU145 (B) cells with transfection of pcDNA3.1 or pcDNA3.1-*MAGI2-AS3*. C. STAT3 reporter activity was detected in PC-3 and DU145 cells with transfection of pcDNA3.1 or pcDNA3.1-*MAGI2-AS3* and treatment of IL6. D. Total STAT3 and p-STAT3 protein levels were measured in PC-3 and DU145 cells with transfection of pcDNA3.1 or pcDNA3.1-*MAGI2-AS3*. **, p<0.01; ***, p<0.001.

To explore how *MAGI2-AS3* regulated cell apoptosis, we detected expression of Bcl-2 and Mcl-1, two key regulators of cell apoptosis, in cells transfected with *MAGI2-AS3*. *MAGI2-AS3* overexpression decreased Bcl-2 and Mcl-1 mRNA in PC-3 and DU145 cells (Fig. 3A-B). Bcl-2 and Mcl-1 were important targets of STAT signaling in prostate cancer. We transfected STAT3 reporter vector into PC-3 cells. As a positive control, IL6 activated STAT3 reporter (Fig. 3C). On the contrary, *MAGI2-AS3* overexpression decreased activity of STAT3 reporter, which was reversed by IL6 administration (Fig. 3C). Furthermore, western blotting showed that *MAGI2-AS3* overexpression decreased both p-STAT3 and total STAT3 protein expression in PC-3 and DU145 cells (Fig. 3D).

### *MAGI2-AS3* and miR-424-5p mutually repressed each other in prostate cancer cells

**Figure 4:**
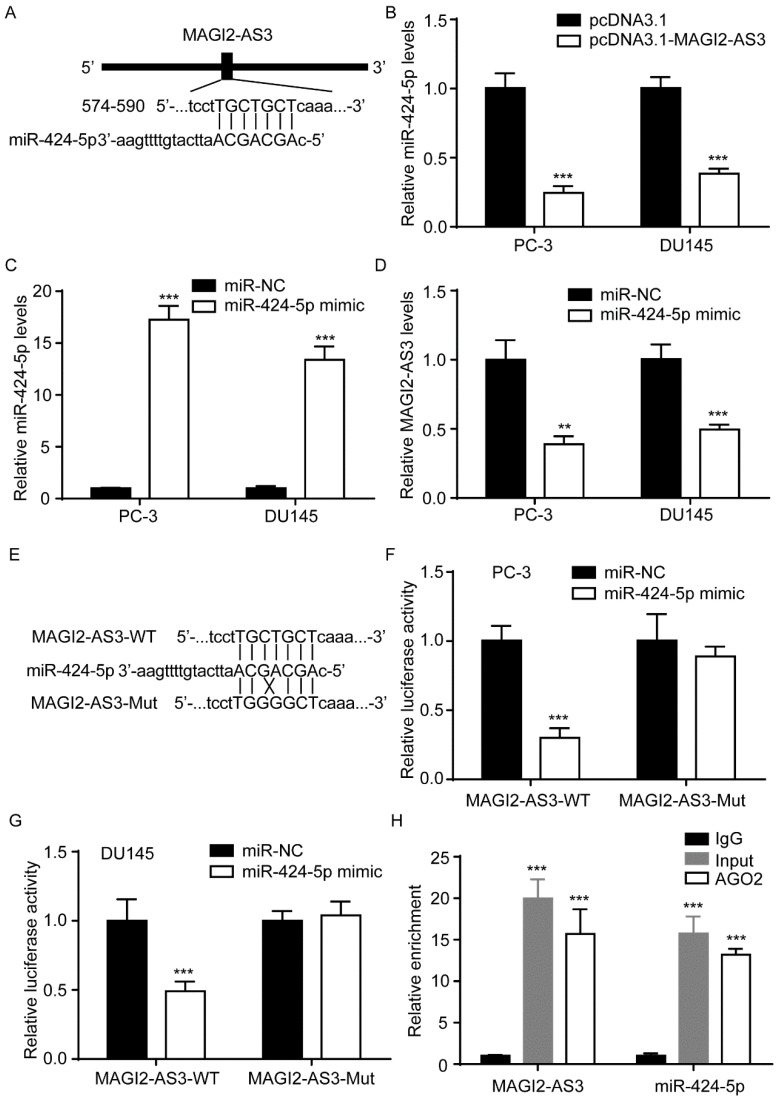
***MAGI2-AS3* regulated miR-424-5p expression in prostate cancer cells.** A. A scheme of binding site for miR-424-5p in *MAGI2-AS3* sequence. B. miR-424-5p expression was detected in PC-3 and DU145 cells with transfection of pcDNA3.1 or pcDNA3.1-*MAGI2-AS3*. C. miR-424-5p expression was detected in PC-3 and DU145 cells with transfection of miR-NC or miR-424-5p mimic. D. *MAGI2-AS3* expression was detected in PC-3 and DU145 cells with transfection of miR-NC or miR-424-5p mimic. E. Sequences of *MAGI2-AS3*-WT and *MAGI2-AS3*-Mut. F-G. The relative luciferase activity was determined in PC-3 (F) and DU145 (G) cells with transfection of miR-NC or miR-424-5p mimic. H. RIP was performed to detect enrichment of *MAGI2-AS3* and miR-424-5p on AGO2. **, p<0.01; ***, p<0.001.

Via bioinformatic analysis, we found a binding site for miR-424-5p in the sequence of *MAGI2-AS3* (Fig. 4A). In PC-3 and DU145 cells, *MAGI2-AS3* overexpression decreased miR-424-5p levels (Fig. 4B). We also transfected miR-424-5p mimic into PC-3 and DU145 cells to upregulate miR-424-5p expression (Fig. 4C). Elevation of miR-424-5p decreased *MAGI2-AS3* expression in PC-3 and DU145 (Fig. 4D). To confirm their direct interaction, *MAGI2-AS3* and the mutation form with two-point mutations were inserted into luciferase reporter vector (Fig. 4E). In PC-3 cells, miR-424-5p mimic repressed luciferase activity in cells transfected with *MAGI2-AS3* not *MAGI2-AS3*-Mut (Fig. 4F). Consistently, similar results were observed in DU145 cells (Fig. 4G). More importantly, RIP assay suggested that *MAGI2-AS3* and miR-424-5p were both enriched in AGO2 antibody (Fig. 4H), indicating their direct interaction.

### *MAGI2-AS3* regulated COP1 to repress STAT3 activity in prostate cancer cells

**Figure 5:**
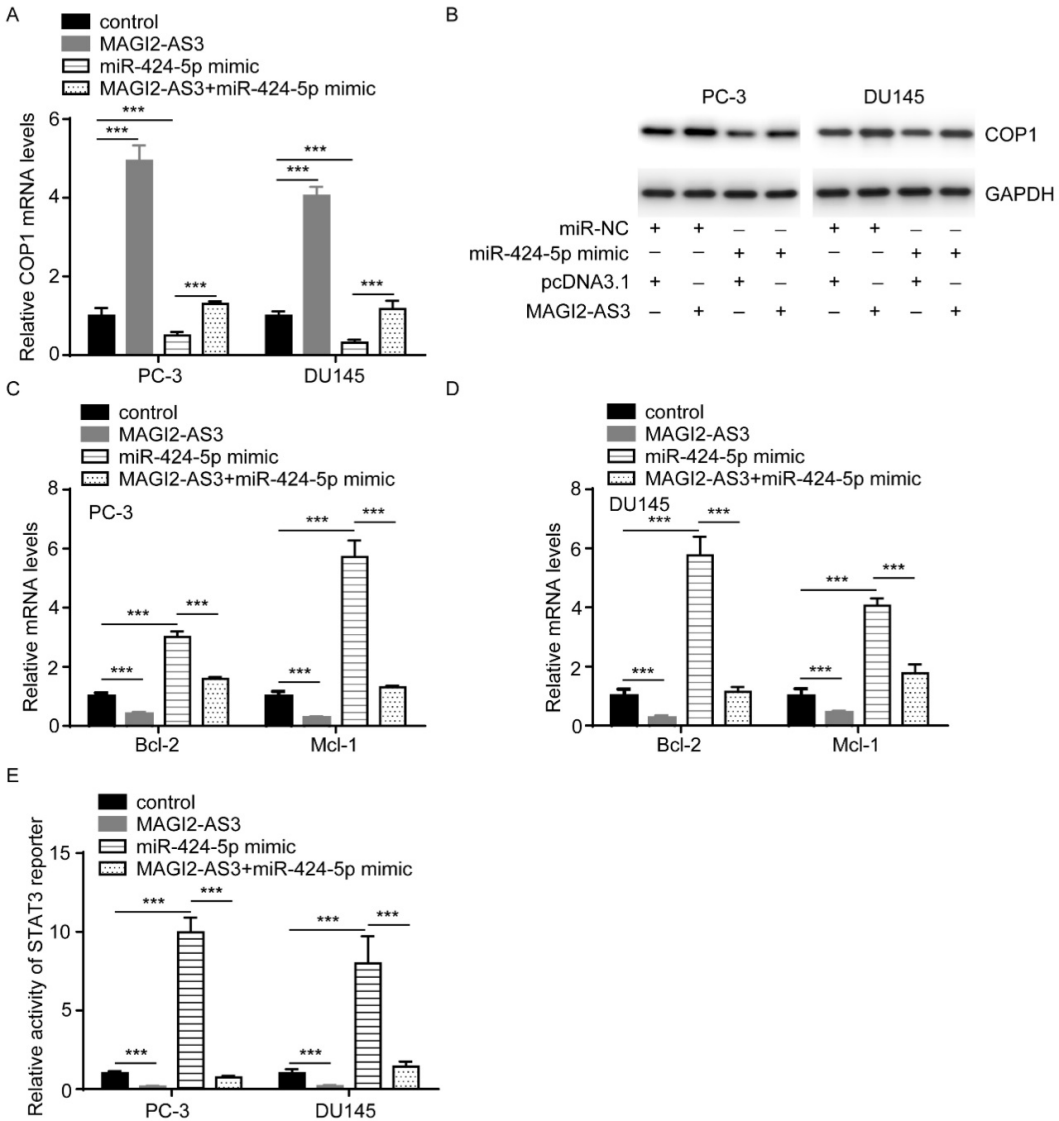
***MAGI2-AS3* inactivated STAT3 signaling by sponging miR-424-5p.** A-B. COP1 mRNA (A) and protein (B) levels were detected in PC-3 and DU145 cells with transfection of pcDNA3.1 or pcDNA3.1-*MAGI2-AS3* and miR-NC or miR-424-5p mimic. C-D. Bcl-2 and Mcl-1 mRNA levels were detected in PC-3 (C) and DU145 (D) cells with transfection of pcDNA3.1 or pcDNA3.1-*MAGI2-AS3* and miR-NC or miR-424-5p mimic. E. STAT3 reporter activity was detected in PC-3 and DU145 cells with transfection of pcDNA3.1 or pcDNA3.1-*MAGI2-AS3* and miR-NC or miR-424-5p mimic. ***, p<0.001.

miR-424-5p regulated STAT3 via targeting COP1 11. We found that COP1 mRNA levels were increased by *MAGI2-AS3*, while decreased by miR-424-5p mimic, in addition, miR-424-5p overexpression reversed the effect of *MAGI2-AS3* on COP1 expression (Fig. 5A). Similarly, western blotting also showed that *MAGI2-AS3* increased COP1 protein expression, meanwhile, miR-424-5p decreased COP1 protein expression and reversed the effect of *MAGI2-AS3* on COP1 protein level (Fig. 5B). Moreover, miR-424-5p mimic elevated Bcl-2 and Mcl-1 mRNA levels and reversed the downregulation of Bcl-2 and Mcl-1 mRNA levels induced by *MAGI2-AS3* in PC-3 and DU145 cells (Fig. 5C-D). Additionally, miR-424-5p could revere the elevation of STAT3 activity by *MAGI2-AS3* overexpression (Fig. 5E).

### *MAGI2-AS3* regulated prostate cancer cell proliferation via inactivation of STAT3 signaling

**Figure 6:**
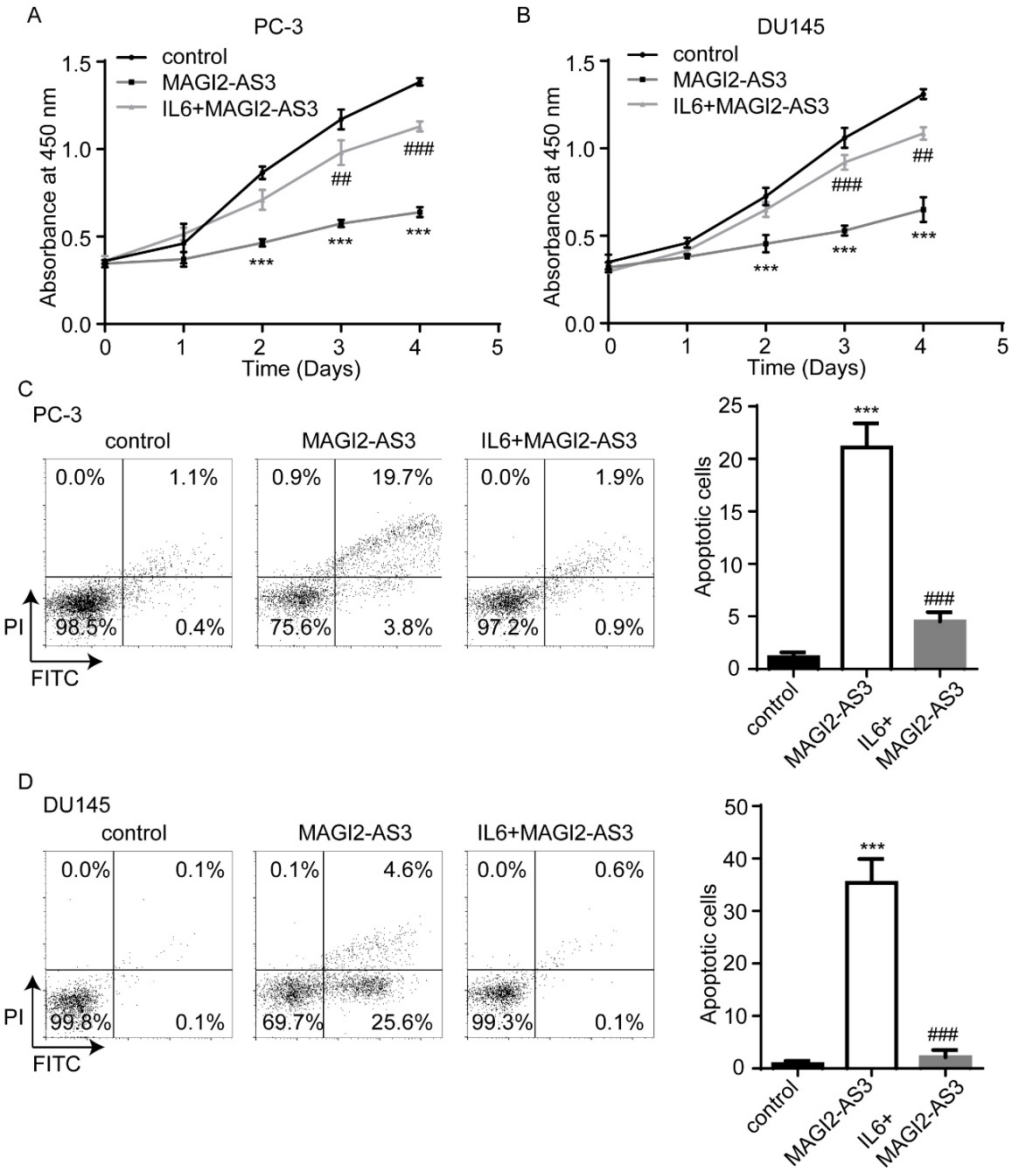
***MAGI2-AS3* regulated prostate cancer cell proliferation via controlling STAT3 activity.** A-B. The cell proliferation ability was detected in PC-3 (A) and DU145 (B) cells with transfection of pcDNA3.1 or pcDNA3.1-*MAGI2-AS3* and treatment of IL6. C-D. The percentage of apoptotic cells was detected in PC-3 (C) and DU145 (D) cells with transfection of pcDNA3.1 or pcDNA3.1-*MAGI2-AS3* and treatment of IL6. *** vs. control group, p<0.001; ### vs. *MAGI2-AS3* group, p<0.001.

To study whether STAT3 signaling was pivotal for the function of *MAGI2-AS3*, we combined IL6 treatment with recombinant *MAGI2-AS3* in PC-3 and DU145 cells. As we expected, IL6 administration attenuated the effect of *MAGI2-AS3* overexpression on cell proliferation and apoptosis (Fig. 6A-D).

### *MAGI2-AS3* was correlated with miR-424-5p, COP1, Bcl-2 and Mcl-1 expression in clinical samples

**Figure 7:**
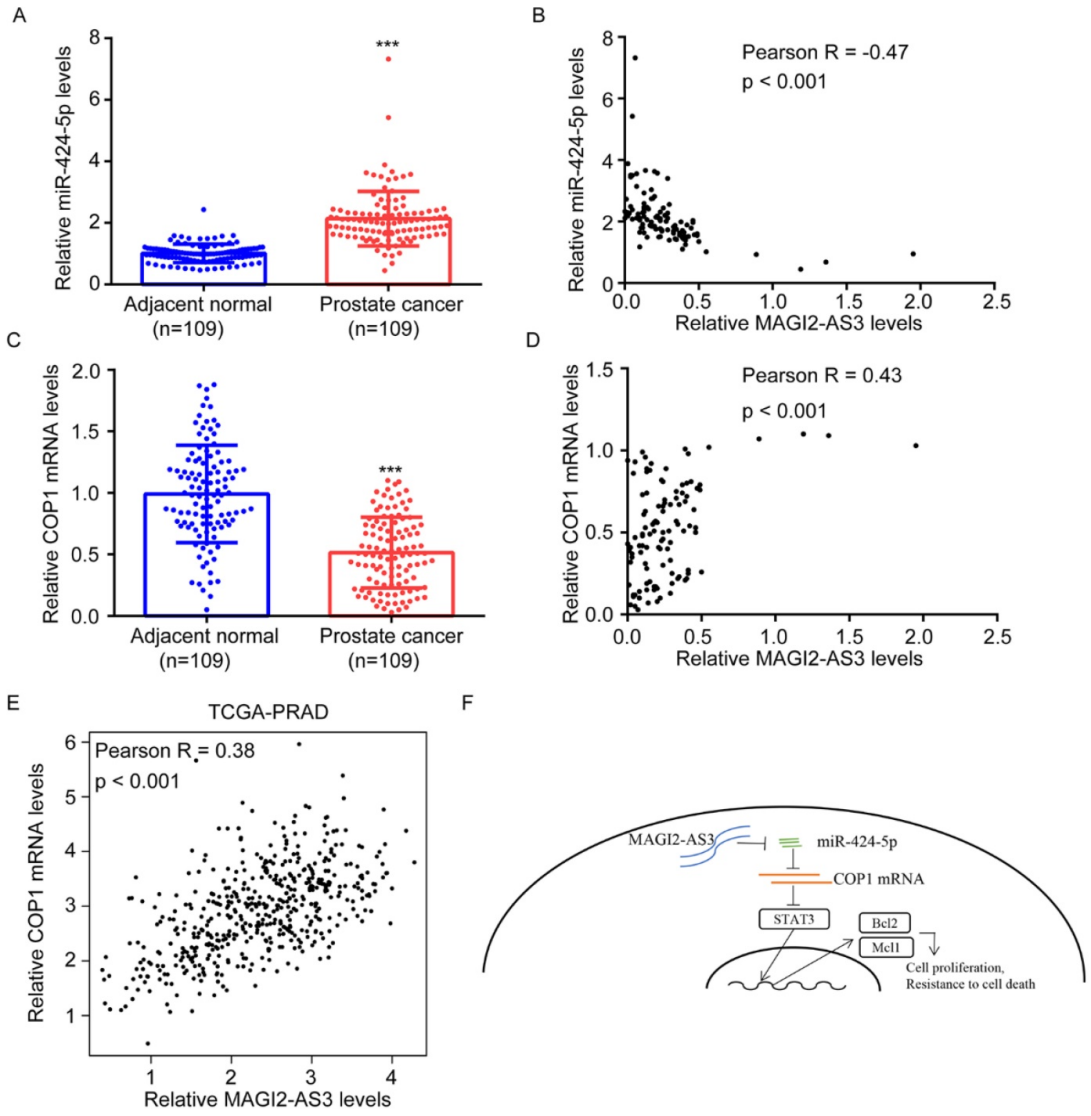
***MAGI2-AS3* expression was associated with miR-424-5p and COP1 expression in clinical samples.** A. miR-424-5p expression was detected in 109 pairs of prostate cancer and adjacent normal prostate by RT-qPCR. B. The association between *MAGI2-AS3* and miR-424-5p levels was analyzed in collected samples by Pearson correlation analysis. C. COP1 mRNA expression was detected in 109 pairs of prostate cancer and adjacent normal prostate by RT-qPCR. D. The associated between *MAGI2-AS3* and COP1 mRNA levels was analyzed in collected samples by Pearson correlation analysis. E. The association between *MAGI2-AS3* and COP1 mRNA levels was analyzed in TCGA data by Pearson correlation analysis. F. A model of the *MAGI2-AS3*/miR-424-5p/COP1 axis in prostate cancer cells. ***, p<0.001.

RT-qPCR showed that miR-424-5p levels were upregulated in collected prostate tumors and its expression was negatively correlated with *MAGI2-AS3* expression (Fig. 7A-B). We further found that COP1 mRNA levels were downregulated in collected prostate tumors, which was positively correlated with *MAGI2-AS3* expression (Fig. 7C-D). In TCGA-PRAD dataset, it was revealed that *MAGI2-AS3* expression was positively correlated with COP1 mRNA levels (Fig. 7E). In conclusion, the current data suggested that *MAGI2-AS3* acted as a sponge for miR-424-5p to elevate COP1 expression and inactivated STAT3 signaling in prostate cancer cells (Fig. 7F).

## Discussion

In recent years, a large number of aberrant expressed lncRNAs have been found to be associated with initiation, uncontrolled cell proliferation, metastasis and drug resistance of cancer 12. In particular, researchers found that several lncRNAs could mediate resistance to cell death to maintain cell proliferation in prostate cancer cells 13, 14. According to WGCNA analysis of datasets (TCGA, GSE17951, GSE7076), Cai *et al*. found that *MAGI-AS3* was one of the downregulated lncRNAs in prostate cancer and was one of prognosis-related genes for patients with prostate cancer 10. In the present study, we also retrieved TCGA and GEO data (GSE46602), consistently, significant downregulation of *MAGI2-AS3* was found. Moreover, we collected patient samples for validation. In consistent with the microarray and RNA sequencing data, RT-qPCR also showed that *MAGI2-AS3* expression was decreased in prostate cancer. However, the role of *MAGI2-AS3* in cancer was controversial. In non-small cell lung cancer, breast cancer and esophageal cancer, *MAGI2-AS3* played a key role in inhibiting cell proliferation and serving as a radiotherapy sensitizer 15-17; on the contrary, *MAGI-AS3* functioned as an oncogenic potential in gastric cancer and colorectal cancer 9, 18. Herein, we found that in prostate cancer cells, forced elevation of *MAGI2-AS3* inhibited prostate cancer cell proliferation and induced cell apoptosis, supporting a tumor suppressor role of *MAGI2-AS3* in prostate cancer.

STAT3 signaling was one of the well-characterized cancer driver pathways in prostate cancer, which was critical for maintaining uncontrolled cell proliferation 19. The activity of STAT3 signaling was tightly controlled by its interactors and several of them were excellent drug targets 20. The hyper-activity of STAT3 signaling was partially due to the dysregulation of non-coding RNAs including lncRNAs in prostate cancer cells 21, 22. The activated STAT3 upregulated Bcl-2, Mcl-1 and several other apoptosis-related genes to mediate resistance to cell apoptosis 23. As we showed in our findings, *MAGI2-AS3* promoted prostate cancer cell apoptosis, and repressed the activity of STAT3 signaling. The findings added new sights into understanding the regulation of STAT3 signaling in prostate cancer.

Many lncRNAs exerted their function via acting as miRNA sponges. Mainly localized in cytoplasm, *MAGI2-AS3* also sponged several miRNAs to regulate cancer progression. It was revealed that *MAGI2-AS3* could sponge miR-141/200a, miR-25 and miR-3163 in different cell backgrounds 9, 16, 18. Similar to *MAGI2-AS3*, miR-424-5p could act as an oncogene or tumor suppressor in different cancer types. miR-424-5p facilitated cancer progression of prostate cancer and inhibited hepatocellular carcinoma development 11, 24. Interestingly, we found a binding site for miR-424-5p in the middle area of *MAGI2-AS3* sequence, we further confirmed that *MAGI2-AS3* and miR-424-5p mutually repressed the expression of each other. Their direct interaction was confirmed by dual luciferase activity assay and RIP assays. miR-424-5p targeted E3 ligase COP1 to stabilize STAT3, thus promoting cell proliferation of prostate cancer cells 11. Our data also showed that COP1 was upregulated and STAT3 was downregulated in prostate cancer cells with overexpression of *MAGI2-AS3*, moreover, the effects of *MAGI2-AS3* on COP1 was reversed by miR-424-5p mimic. These results indicated that *MAGI2-AS3* activated STAT3 signaling via sponge miR-424-5p in prostate cancer cells.

Collectively, the current study defined *MAGI2-AS3* as a novel tumor suppressor in prostate cancer and suggested *MAGI2-AS3* as a biomarker and drug target for the patients with prostate cancer.

## Figures and Tables

**Table 1:** Association between MAGI2-AS3 levels and the clinicopathological features of patients with prostate cancer

Characteristics	Number of cases		p value
MAGI2-AS3 expression	High	Low	
	55	54	
Age (years)			0.435
≤60	19	23	
>60	36	31	
Gleason score			0.005
2-7	47	33	
8-10	8	21	
Tumor stage			0.441
T2	34	29	
T3-4	21	25	

## References

[1] Siegel RL, Miller KD, Jemal A (2018). Cancer statistics, 2018. CA: A Cancer Journal for Clinicians.

[2] Du Z, Sun T, Hacisuleyman E, Fei T, Wang X, Brown M (2016). Integrative analyses reveal a long noncoding RNA-mediated sponge regulatory network in prostate cancer. Nat Commun.

[3] Lin JZ, Wang WW, Hu TT, Zhu GY, Li LN, Zhang CY (2020). FOXM1 contributes to docetaxel resistance in castration-resistant prostate cancer by inducing AMPK/mTOR-mediated autophagy. Cancer Lett.

[4] Yu Y, Gao F, He Q, Li G, Ding G (2020). lncRNA UCA1 Functions as a ceRNA to Promote Prostate Cancer Progression via Sponging miR143. Mol Ther Nucleic Acids.

[5] Wu M, Huang Y, Chen T, Wang W, Yang S, Ye Z (2019). LncRNA MEG3 inhibits the progression of prostate cancer by modulating miR-9-5p/QKI-5 axis. J Cell Mol Med.

[6] Hua JT, Chen S, He HH (2019). Landscape of Noncoding RNA in Prostate Cancer. Trends Genet.

[7] Tian T, Gong Z, Wang M, Hao R, Lin S, Liu K (2018). Identification of long non-coding RNA signatures in triple-negative breast cancer. Cancer Cell Int.

[8] Zhu N, Hou J, Wu Y, Liu J, Li G, Zhao W (2018). Integrated analysis of a competing endogenous RNA network reveals key lncRNAs as potential prognostic biomarkers for human bladder cancer. Medicine (Baltimore).

[9] Li D, Wang J, Zhang M, Hu X, She J, Qiu X (2020). LncRNA *MAGI2-AS3* Is Regulated by BRD4 and Promotes Gastric Cancer Progression via Maintaining ZEB1 Overexpression by Sponging miR-141/200a. Mol Ther Nucleic Acids.

[10] Cai J, Chen Z, Chen X, Huang H, Lin X, Miao B (2020). Coexpression Network Analysis Identifies a Novel Nine-RNA Signature to Improve Prognostic Prediction for Prostate Cancer Patients. Biomed Res Int.

[11] Dallavalle C, Albino D, Civenni G, Merulla J, Ostano P, Mello-Grand M (2016). MicroRNA-424 impairs ubiquitination to activate STAT3 and promote prostate tumor progression. J Clin Invest.

[12] Wei L, Wang X, Lv L, Zheng Y, Zhang N, Yang M (2019). The emerging role of noncoding RNAs in colorectal cancer chemoresistance. Cell Oncol.

[13] Misawa A, Takayama K, Urano T, Inoue S (2016). Androgen-induced Long Noncoding RNA (lncRNA) SOCS2-AS1 Promotes Cell Growth and Inhibits Apoptosis in Prostate Cancer Cells. J Biol Chem.

[14] Zhu Y, Tong Y, Wu J, Liu Y, Zhao M (2019). Knockdown of LncRNA GHET1 suppresses prostate cancer cell proliferation by inhibiting HIF-1α/Notch-1 signaling pathway via KLF2. Biofactors.

[15] Xu X, Yuan X, Ni J, Guo J, Gao Y, Yin W (2021). *MAGI2-AS3* inhibits breast cancer by downregulating DNA methylation of MAGI2. J Cell Physiol.

[16] Sui Y, Chi W, Feng L, Jiang J (2020). LncRNA *MAGI2-AS3* is downregulated in non-small cell lung cancer and may be a sponge of miR-25. BMC Pulm Med.

[17] Cheng W, Shi X, Lin M, Yao Q, Ma J, Li J (2020). LncRNA *MAGI2-AS3* Overexpression Sensitizes Esophageal Cancer Cells to Irradiation Through Down-Regulation of HOXB7 via EZH2. Front Cell Dev Biol.

[18] Ren H, Li Z, Tang Z, Li J, Lang X (2020). Long noncoding *MAGI2-AS3* promotes colorectal cancer progression through regulating miR-3163/TMEM106B axis. J Cell Physiol.

[19] Carpenter RL, Lo HW (2014). STAT3 Target Genes Relevant to Human Cancers. Cancers (Basel).

[20] Laudisi F, Cherubini F, Monteleone G, Stolfi C (2018). STAT3 Interactors as Potential Therapeutic Targets for Cancer Treatment. Int J Mol Sci.

[21] Luo J, Wang K, Yeh S, Sun Y, Liang L, Xiao Y (2019). LncRNA-p21 alters the antiandrogen enzalutamide-induced prostate cancer neuroendocrine differentiation via modulating the EZH2/STAT3 signaling. Nat Commun.

[22] Wang N, Jiang Y, Lv S, Wen H, Wu D, Wei Q (2020). HOTAIR expands the population of prostatic cancer stem-like cells and causes Docetaxel resistance via activating STAT3 signaling. Aging (Albany NY).

[23] Hu H, Lee HJ, Jiang C, Zhang J, Wang L, Zhao Y (2008). Penta-1,2,3,4,6-O-galloyl-beta-D-glucose induces p53 and inhibits STAT3 in prostate cancer cells in vitro and suppresses prostate xenograft tumor growth in vivo. Mol Cancer Ther.

[24] Teng F, Zhang JX, Chang QM, Wu XB, Tang WG, Wang JF (2020). LncRNA MYLK-AS1 facilitates tumor progression and angiogenesis by targeting miR-424-5p/E2F7 axis and activating VEGFR-2 signaling pathway in hepatocellular carcinoma. J Exp Clin Cancer Res.

